# replicateFest: an R package and shiny app for analysis of T cell receptor repertoire data from the functional expansion

**DOI:** 10.3389/fimmu.2026.1937788

**Published:** 2026-10-02

**Authors:** Ludmila Danilova, Alexander V. Favorov, Kellie N. Smith, Leslie Cope

**Affiliations:** 1 Department of Oncology, Sidney Kimmel Comprehensive Cancer Center, Johns Hopkins School of Medicine, Baltimore, MD, United States; 2 Bloomberg-Kimmel Institute for Cancer Immunotherapy, Johns Hopkins School of Medicine, Baltimore, MD, United States

**Keywords:** cancer immunotherapy, data analyze, functional expansion, R package, T cell repertoire

## Abstract

**Motivation:**

The Functional Expansion of Specific T cell (FEST)-based assays combine short-term peptide stimulation with TCR sequencing to identify clonotypes that expand in response to specific antigens. These approaches have proven invaluable for detecting antigen-specific T cell responses, guiding vaccine development, and assessing checkpoint blockade efficacy. However, variability introduced by biological and technical replicates poses challenges for reproducibility and interpretation, and existing computational tools do not address replicate-level analysis in these assays.

**Results:**

We developed replicateFest, a computational framework implemented as an R package and Shiny web application, to analyze FEST-based TCR-seq data with and without replicates. replicateFest applies Fisher’s exact test for non-replicate datasets and negative binomial modeling for replicate experiments, returning adjusted p-values and odds ratios to identify clonotypes significantly expanded in antigen-stimulated conditions. The framework distinguishes FEST-expanded clonotypes (relative to a no-antigen control) and FEST-positive clonotypes (expanded compared to all other conditions). Validation using *in silico* datasets confirmed accurate detection of antigen-specific clonotypes. Application to published HIV-1 epitope stimulation data reproduced original findings and demonstrated replicateFest’s utility for reproducibility assessment and quality control.

## 1 Introduction

Monitoring antigen-specific T cell responses has become a cornerstone of cancer immunology and immunotherapy research. The ability to detect and quantify clonal expansion of T cells in response to various classes of antigens provides critical insights into the dynamics of T cell mediated immunity and the effectiveness of therapeutic interventions. Traditional approaches, such as ELISPOT or intracellular cytokine staining, offer functional readouts but lack the resolution to capture the full diversity and clonality of T cell responses.

Recent advances in high-throughput T cell receptor sequencing (TCR-seq) have transformed our capacity to characterize T cell repertoires at unprecedented depth. By coupling TCR-seq with antigen stimulation assays, researchers can identify clonotypes that expand in response to specific peptides, enabling precise mapping of antigen-specific T cell populations. The Functional Expansion of Specific T cells (FEST) family of assays exemplifies this approach by integrating short-term peptide stimulation with TCR-seq to detect antigen-specific clonotypes. These assays use peptides representing candidate antigens from viruses (ViraFEST), from tumors (tumor-associated antigens, TAAFEST), or from mutations (mutation-associated neoantigens, MANAFEST). The latter was previously published by our group (1) and incorporated a bioinformatics workflow for identifying antigen-responsive clonotypes from FEST-based TCR-seq data generated without biological replicates. As FEST assays have expanded to include replicate experimental designs, additional statistical approaches are needed to account for biological variability and improve confidence in the identification of antigen-responsive clonotypes.

To our knowledge, replicateFest is the first publicly available computational framework specifically designed for the analysis of FEST-based antigen stimulation experiments. Unlike existing methods that infer antigen specificity from repertoire structure or TCR sequence similarity, replicateFest directly evaluates antigen-driven clonal expansion by comparing stimulated and control cultures using statistical models tailored to the presence or absence of replicates. The framework includes both an R package and an interactive Shiny application, providing a standardized and reproducible workflow for analysis of MANAFEST, ViraFEST, TAAFEST, and related functional expansion assays. By explicitly supporting replicate-aware statistical testing while maintaining compatibility with non-replicate experimental designs, replicateFest enhances the reliability of antigen-responsive clonotype identification in FEST-based TCR-seq studies.

## 2 Methods

### 2.1 The replicateFest framework

Our analysis framework is designed to analyze TCR-seq data generated from a specific experimental setup. A typical experiment involved T cells obtained from a patient and cultured *in vitro* under multiple antigen conditions. These conditions included stimulation with peptides of interest, such as mutation-associated neoantigens (MANAs), viral epitopes, or other antigens, as well as a control condition without peptide to evaluate non-specific T cell expansion. After several days of culture (depending on the experimental design), DNA is extracted for TCR Vβ CDR3 sequencing. Such experiments have been previously described in (1–3).

The replicateFest data analysis workflow is depicted on **Figure 1** and consists of the following main steps: importing the FEST-based TCR-seq data, estimating clone abundance, and filtering the results to select significantly expanded clones. Users can perform the analysis online using Shiny application with standardized model parameters and filtration criteria (**Figure 2**), while a free R package offers the experienced user the ability to customize analysis, filtration, and output formats.

**Figure 1:**
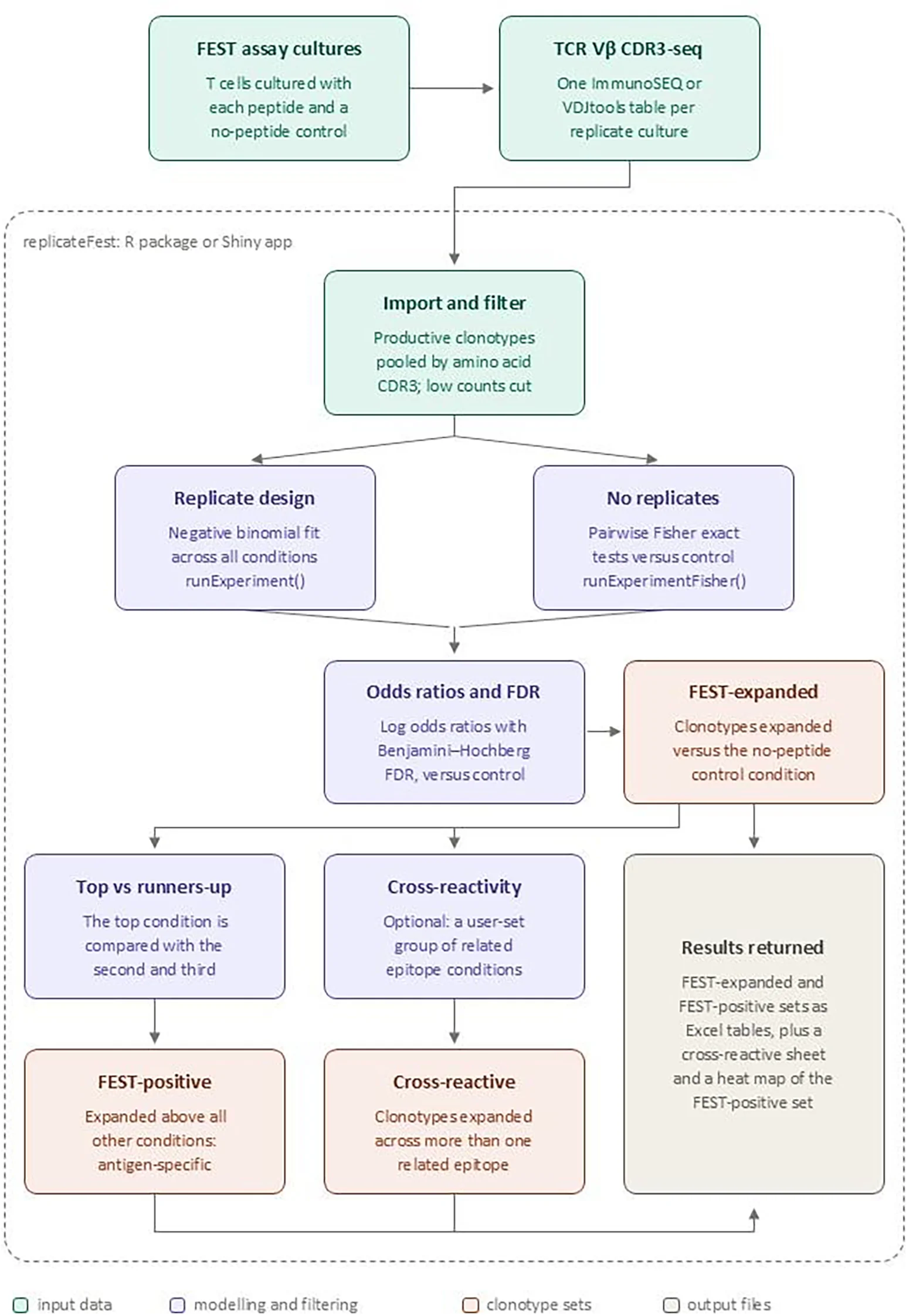
Analysis workflow of the replicateFest framework. T cells are cultured in parallel under one condition per peptide of interest together with a no-peptide control condition, with biological or technical replicates where the design allows, and the TCR Vβ CDR3 region is sequenced for every culture (green). The resulting per-sample tables, e.g. in Adaptive ImmunoSEQ or VDJtools format, are the input to replicateFest, which can be run either as an R package or through the accompanying Shiny application (dashed outline). At import, only productive clonotypes are retained, nucleotide sequences translating into the same amino acid CDR3 are aggregated, and clonotypes with low template counts are discarded. Clonotype abundance is then estimated in one of two ways depending on the design (purple): a negative binomial model fitted across all conditions simultaneously when replicates are available (runExperiment), or pairwise Fisher’s exact tests against the control when they are not (runExperimentFisher). Both routes yield an odds ratio and a Benjamini–Hochberg adjusted p-value for every clonotype in every condition relative to the no-peptide control. Clonotypes passing the odds ratio and FDR thresholds constitute the FEST-expanded set (orange). This set is refined along two independent branches: clonotypes whose top condition is significantly above the next-ranked conditions are reported as FEST-positive, that is, antigen-specific; optionally, clonotypes expanded across a user-defined group of related epitope conditions are reported as cross-reactive. Both clonotype sets and the cross-reactive set are written out as Excel tables; additionally, a heat map of the FEST-positive clonotypes can be saved as a PDF file (grey).

**Figure 2:**
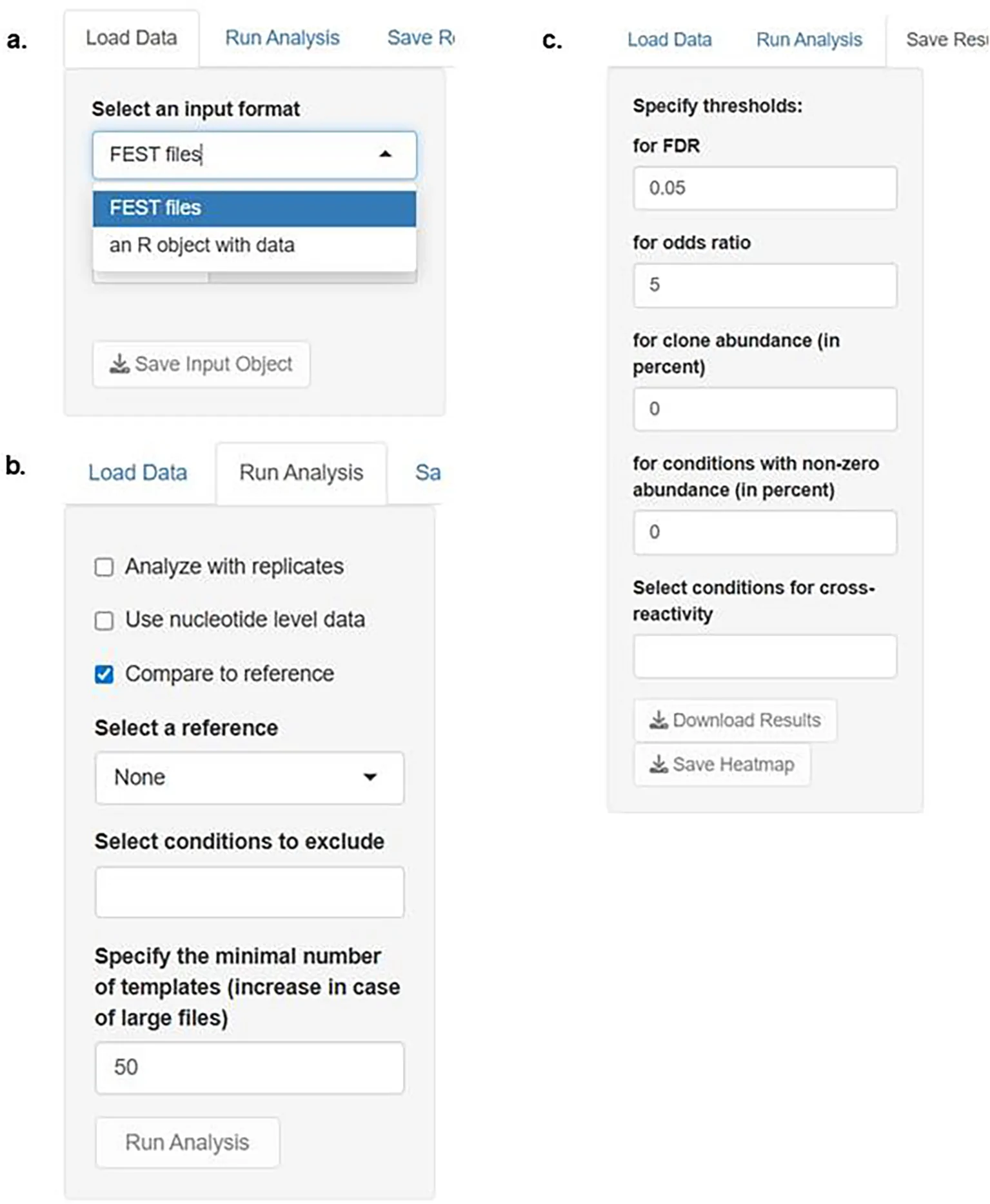
Screenshots of Shiny web application that demonstrate three main steps of the analysis: Load Data **(a)**, Run Analysis **(b)**, Save Results **(c)**. **(a)** The ‘Load Data’ tab allows users to upload FEST files or a previously saved R object with the data. **(b)** After the data is successfully loaded, users should select the ‘Analyze with replicates’ checkbox if the loaded data contain replicates and select the reference condition on the ‘Run Analysis’ tab. Additionally, users can specify conditions that should be excluded from the analysis and the minimal number of templates (reads). After that, click the ‘Run Analysis’ button to perform the analysis with the specified parameters. **(c)** After the analysis is done, the user can select parameters to save the results on the ‘Save Results’ tab. The results from the same analysis can be saved with different thresholds for clones to be FEST-expanded and FEST-positive, such as FDR, OR, clone abundances, and the percent of conditions with non-zero abundances.

#### 2.1.1 Importing data

By default, replicateFest accepts tab-delimited files as exported from the Adaptive Biotechnologies ImmunoSEQ platform or VDJtools. For the analysis, file names should follow the format ‘sampleID_condition_replicate.tsv ‘, e.g., ‘sample1_HIV_1.txt’. During import, templates are prefiltered to include only productive clonotypes, and nucleotide sequences translating into the same amino acid sequence are aggregated. The user has an option to filter out templates with low counts to reduce the number of clonotypes included in downstream analysis and improve runtime.

#### 2.1.2 Estimating clone abundance

Our previously published workflow for unreplicated FEST experiments used odds ratios to estimate levels of clonal expansion compared to no-peptide or other control, and evaluated significance with pairwise Fisher’s exact tests. In case of data with experimental replicates, clonotype abundance was modeled using a negative binomial distribution to account for overdispersion commonly observed in sequencing count data. The model compares antigen-stimulated and reference conditions while borrowing information across clonotypes through empirical dispersion estimation. Clonotypes that failed to meet the minimum abundance threshold were excluded prior to model fitting. As with other count-based approaches, statistical power may be reduced for extremely sparse clonotypes, low sequencing depth, or experiments with a limited number of replicates. replicateFest does not explicitly model biological processes such as T cell expansion kinetics, clonal competition, or sampling effects arising during cell culture and sequencing. For each clone, we fit a negative binomial model to estimate the abundances for all culture conditions simultaneously. The coefficients in the negative binomial model are interpreted as log odds ratios calculated relative to a no-peptide control. The analysis returns p-values adjusted by Benjamini-Hochberg multiple test correction (FDR).

#### 2.1.3 Filtering for significant and unique expansions

The software offers several criteria for selecting *FEST-expanded clonotypes* relative to the reference: (i) expanded in a condition of interest compared with the reference at an FDR less than the specified threshold, and (ii) having a positive odds ratio above the specified threshold. The default significance threshold is FDR< 0.05; however, this parameter can be adjusted by the user according to the goals of a particular study. More stringent thresholds (e.g., FDR< 0.01) may be used when higher specificity is desired or when consistency with previously published analyses is required. Additionally, we identify a subset of FEST-expanded clonotypes that are expanded in the condition of interest compared with all other conditions tested in parallel; these are designated *FEST-positive clonotypes*. Both sets (FEST-expanded and FEST-positive) are saved in output Excel tables, and FEST-positive clonotypes are visualized in a heatmap.

#### 2.1.4 Implementation and availability

The replicateFest computational framework is implemented as an R package (R ≥ 4.0) and a Shiny application, providing an interactive interface for non-programmatic users. Both components are distributed under the Apache-2.0 license and are freely available at https://github.com/OncologyQS/replicateFest, and the Shiny application is hosted at http://www.stat-apps.onc.jhmi.edu/FEST/.

The R package supports large-scale analysis of multiple input datasets through two main functions: runExperiment for data with replicates and runExperimentFisher for data without replicates. The Shiny web application provides interactive analysis and result export to Excel, making the workflow accessible to users without programming experience.

### 2.2 *In silico* dataset generation

The replicateFest R package includes a set of standardized tests based on artificial data, to ensure the integrity of the code itself and interactions with dependencies. Briefly, synthetic T-cell receptor repertoire data were generated by randomly sampling amino acid CDR3 sequences from the 20 standard residues with lengths between 12 and 16, while nucleotide sequences were generated from the four canonical bases with lengths between 36 and 45. The background counts of clonotypes were randomly selected to be around 10. To simulate peptide-driven clonal expansion, a single clonotype in each stimulated sample was replaced with a clone that expanded about 100 times from the background. Details can be found in the R package.

### 2.3 Analysis of HIV-1 data

We used the T cell sequencing dataset with replicates that were previously published in a study of HIV-1 epitope variants by Chen et al. (3). The data were downloaded from the Adaptive Biotechnologies ImmuneACCESS Database, https://clients.adaptivebiotech.com/pub/chan-2020-fi. We analyzed the dataset in the R programming environment (v4.6.0) and followed the analysis performed in the Chen et al. study as closely as possible. We excluded samples with fewer than 5,000 productive reads, leaving 79 samples of 25 peptide conditions and the reference (no peptide) condition for the downstream analysis. Then we ran replicateFest (v0.1.0) with the cross-reactive option enabled, 0.01 for the FDR threshold, and 5 for the OR threshold to match the thresholds used in the Chen et al. study. The output of the analysis is presented in the **Supplementary Table 1**.

### 2.4 Simulation study

#### 2.4.1 Data generation

Observed T-cell clone count data were drawn from 79 samples from Chen et al. (3). We removed clones found to be FEST-expanded (**Supplementary Table 1**), as well as clones with fewer than 50 total reads, leaving 351 clones with positive counts but no significant expansion. From the dataset, we selected 10 peptide conditions with three replicates and the reference (no peptide) condition (n = 33). For each clone, the mean and variance of counts were calculated across all experimental conditions (**Figure 3**), fitting a loess line to model variance as a function of mean.

**Figure 3:**
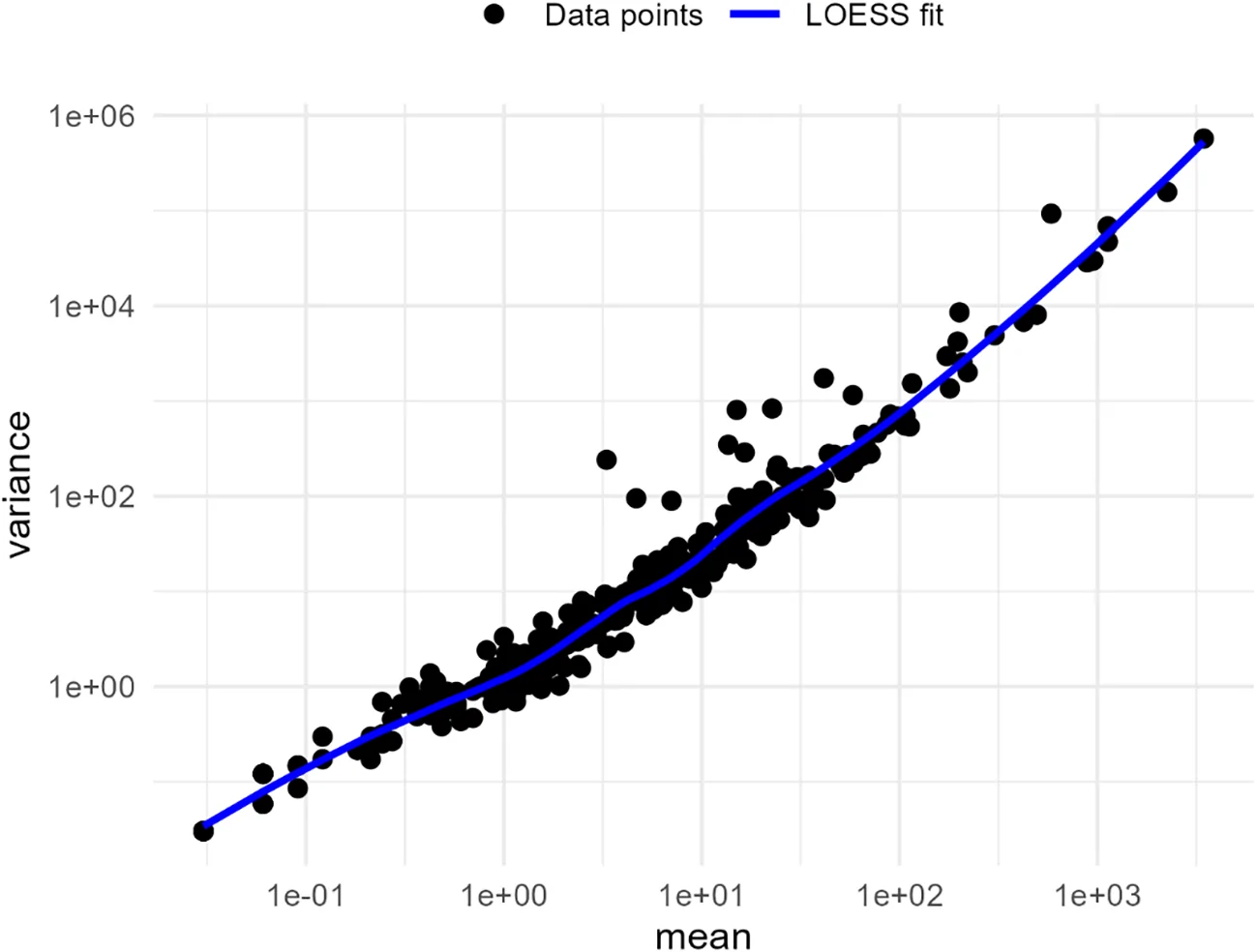
The mean and variance of counts of 351 clonotypes calculated across 33 samples. A loess-fitted line is shown in blue.

Simulated clones were generated across a 3 × 3 factorial design defined by three starting frequency levels (L1, L2, L3; corresponding to the 50th, 75th, and 95th percentiles of the empirical mean distribution) and three expansion factors (E1 = 2×, E2 = 4×, E3 = 8×) that yielded nine parameter combinations. The counts were drawn from a negative binomial distribution parameterized by a mean (*μ*) and dispersion (size) parameter, where $\text{size}=\mu^{2}/\left(\sigma^{2}-\mu \right)$. For non-expanded replicates, the dispersion was derived from the empirical mean–variance relationship at the clone’s baseline mean. For expanded replicates, the dispersion was interpolated using an Akima spline fit to the loess line.

Each parameter combination was used to simulate 10 clones, yielding a matrix of 90 simulated clones (10 clones × 9 combinations). Each clone was expanded in three replicates of one condition. Simulated clone counts were appended to the observed background count matrix to produce complete count matrices. This procedure was repeated 10 times to generate 10 independent simulated datasets.

#### 2.4.2 Application of replicateFest

Each of the 10 simulated datasets was analyzed using replicateFest (v0.1.0), with the no-peptide condition (NoPeptide) specified as the reference condition and the default thresholds (FDR< 0.05, OR > 1). For 90 simulated clones in each dataset, clone-level results were tabulated in a 3 × 3 contingency table (frequency level × expansion level), allowing assessment of sensitivity across all simulated datasets. Detection rates were summarized across 10 simulated datasets as the mean and standard deviation of the proportions of correctly identified FEST-expanded clones out of simulated clones within each cell of the 3 × 3 factorial design (**Table 1**; **Figure 4**).

**Table 1:** Nine clones that are reported in the study Chen et al.

Clone	HIV protein	Is FEST-expanded?	Is cross-reactive?	Is reported as cross-reactive in Chen et al.?
CASSPGVGNTEAFF	Gag	TRUE	TRUE	TRUE
CASSLDPGANTEAFF	Gag	TRUE	FALSE	TRUE
CASSPRQAGLVTQYF	Gag	TRUE	FALSE	FALSE
CASSPRWGDAGELFF	Nef	TRUE	TRUE	TRUE
CAWETGVRDGYTF	Nef	TRUE	FALSE	FALSE
CASSLLAGGSLDEQFF	Nef	TRUE	TRUE	TRUE
CASSLDLRTFTYEQYF	Nef	TRUE	TRUE	TRUE
CASSLERVGYNEQFF	Nef	TRUE	TRUE	FALSE
CAISLMGTEAFF	Nef	TRUE	FALSE	FALSE

**Figure 4:**
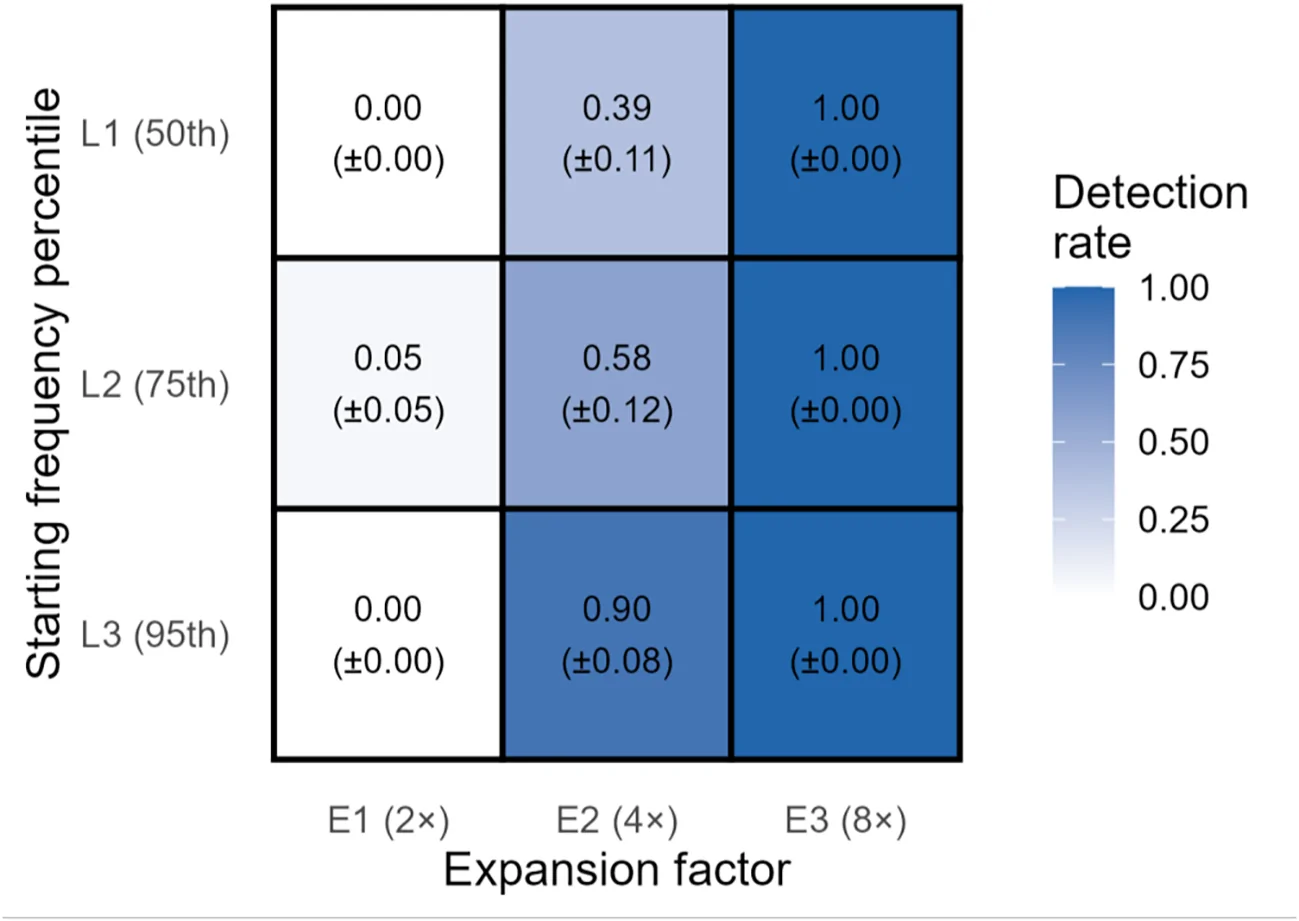
The results of the methods sensitivity under controlled conditions. The results summarized as mean detection rate (± SD) across 10 simulations. Each simulation contained clones with three starting frequency levels (L1, L2, L3; corresponding to the 50th, 75th, and 95th percentiles of the empirical mean distribution) and three expansion factors (E1 = 2×, E2 = 4×, E3 = 8×).

## 3 Results

### 3.1 *In silico* experimental data

To evaluate the framework in a controlled setting, we generated *in silico* experimental datasets with and without replicates (see details in Methods). Each simulated experiment included five peptide conditions, with only one clonotype expanded per condition, and one condition without expansion. replicateFest successfully identified both FEST-expanded and FEST-positive clonotypes in experiments with and without replicates. These *in silico* datasets and corresponding results are included as test data within the R package. Overall, these results demonstrate that replicateFest reliably detects clonal expansion under well-defined conditions and provides consistent performance regardless of the presence of experimental replicates.

### 3.2 Application to HIV-1 data

We applied our framework to previously published data that was used to identify cross-reactive recall CD8^+^ T cell clonotypes that respond to autologous HIV-1 epitope variants from the study by Chen et al. (3). The study demonstrated that memory CD8^+^ T cells can recognize multiple epitope variants, highlighting the breadth and adaptability of antiviral T cell immunity. By leveraging TCR-seq, the authors assessed 25 peptides representing epitope variants of Gag and Nef HIV proteins to evaluate T cell response to stimuli. The study adapted non-replicated version of the MANAFEST assay (2) to the replicated dataset by applying additional filtering criteria. After applying the criteria, the authors reported nine T cell clones that significantly expanded in response to stimuli (three clones in response to Gag epitopes and six clones in response to Nef epitopes), where five of them were reported as cross-reactive (**Table 1**).

To validate reproducibility and assess replicate-level consistency, replicateFest was applied to the TCR-seq data from the HIV-1 study, trying to recapitulate the analysis as closely as possible (see details in Methods). We excluded samples that were excluded in the study, and applied similar thresholds, specifically, for FDR less than 0.01 and OR more than 5 (**Supplementary Table 1**). We were able to successfully identify all nine clonotypes reported in the study as FEST-expanded. Additionally, we identified four out of five cross-reactive clonotypes, and one cross-reactive clonotype that was not reported as cross-reactive by Chen et all. These minor discrepancies were due to the different methodology of calling FEST-expanded clonotypes by Chen et al. and replicateFest. These results demonstrate the ability of replicateFest to reliably find antigen-specific expanded and cross-reactive clonotypes in real experimental data.

### 3.3 Application to the simulated data

To evaluate replicateFest performance, we simulated clones with known background levels and expansion rates from a negative binomial distribution, using empirical count data as a basis for realistic parameterization (see details in Methods). We used a 3 × 3 factorial design with three starting frequency levels (L1, L2, L3; corresponding to the 50th, 75th, and 95th percentiles of the empirical mean distribution) and three expansion factors (E1 = 2×, E2 = 4×, E3 = 8×). We generated 10 simulated datasets with 90 expanded clonotypes corresponding to nine combinations of the factorial design (ten clonotypes for each combination), and analyzed these datasets with replicateFest under default parameters. After that, we evaluated how many clonotypes out of 90 were FEST-expanded across 10 simulated datasets (**Figure 4**) and calculated the mean and standard deviation of the detection rate. At the lowest expansion level (E1, 2×), detection was near zero across all starting frequency levels, indicating that 2-fold expansions fall below the method’s detection threshold under the simulated conditions. At moderate expansion (E2, 4×), sensitivity increased substantially with starting clone frequency, ranging from 0.42 ± 0.10 at L1 to 0.92 ± 0.10 at L3, with the greatest variability across replicates observed in this regime. At the highest expansion level (E3, 8×), detection was near-perfect regardless of starting frequency (≥ 0.98 in all cases), with no variability at L3. Overall, these results indicate that replicateFest exhibits a clear dependence on both clonal abundance and expansion magnitude, with robust detection achieved primarily at higher expansion levels and frequencies, thereby defining the practical sensitivity limits of the method under realistic repertoire conditions.

## 4 Discussion

The ability to accurately monitor antigen-specific T cell responses is critical for understanding antitumor immunity and guiding immunotherapy strategies. FEST-based assays, including MANAFEST, have enabled sensitive detection of clonotype expansions following antigen stimulation by leveraging TCR-seq. However, the complexity of TCR repertoires and variability introduced by biological and technical replicates present significant challenges for reproducibility and interpretation.

Here we introduce replicateFest, a computational framework comprising an R package and an interactive Shiny application to address these challenges by providing a unified computational framework for analyzing FEST-based TCR-seq data. By incorporating statistical models tailored to experimental design – Fisher’s exact test for single-replicate data and negative binomial modeling for replicate datasets – replicateFest ensures robust identification of antigen-specific clonotypes. Although replicateFest was validated using *in silico* data and a previously published HIV-1 dataset, the framework was designed to operate on clonotype count data generated from the broader family of FEST assays and is therefore applicable to neoantigen, viral, tumor-associated antigen, and vaccine-response studies. The negative binomial regression model is independent of antigen type, relying only on clonotype abundance measurements across experimental conditions, so we expect the framework to generalize across FEST-based experimental settings.

The replicateFest framework is distinct from existing computational tools developed to identify antigen-responsive TCRs from stimulation-based assays by detecting patterns of sequence convergence and clonal enrichment. These include ALICE (Antigen-specific Lymphocyte Identification by Clustering of Expanded) (4), TCRNET (a TCR neighbor enrichment test) (5), which employs a network-based framework to identify neighboring TCRs connected with similar CDR3 sequences, as well as GLIPH2 (6), tcrdist3 (7), and TCRscape (8) none of which were designed for FEST-based peptide-stimulation experiments.

Successful testing on the *in silico* datasets and validation on the previously published HIV-1 epitope stimulation data demonstrate that replicateFest reliably identifies expanded clonotypes and reproduces findings from complex experimental designs. Additionally, the simulation results provide important insights into the sensitivity and operational limits of the replicateFest framework under realistic repertoire conditions. Detection performance was strongly influenced by both the baseline abundance of clonotypes and the magnitude of expansion, consistent with theoretical expectations for count-based sequencing data. In particular, the near absence of detection at 2-fold expansion indicates that modest antigen-driven changes may be difficult to distinguish from background variability, especially for low-frequency clones. In contrast, the marked improvement at 4-fold expansion and the near-perfect detection observed at 8-fold expansion demonstrate that replicateFest is highly effective in identifying robust antigen-specific signals when clonal expansion exceeds the noise threshold inherent to TCR-seq data. The increased variability observed at intermediate expansion levels further highlights the stochastic nature of sampling and the importance of replicate-aware modeling. Collectively, these findings emphasize that both clonal frequency and expansion magnitude are critical determinants of detection power and underscore the value of incorporating experimental replicates to improve confidence in identifying true antigen-responsive T cell populations. It should be noted that neither the simulation nor the analysis of previously published HIV-1 data supports an examination of specificity. Genuinely expanded clonotypes may not have achieved statistical significance in prior analyses. The current study focused on providing a practical statistical framework for FEST-based TCR-seq experiments rather than modeling the biological processes that generate repertoire data. Consequently, the *in silico* datasets used for validation did not explicitly incorporate antigen-driven expansion kinetics, clonal competition, repertoire evolution, sampling effects, or sequencing-depth variation. Previous studies have demonstrated the importance of such factors in shaping observed TCR repertoires, including models of T cell expansion dynamics (9), clonal competition and repertoire evolution (10), and the impact of sequencing depth and sampling variability on clonotype detection (11). Future work will evaluate replicateFest using simulation frameworks that incorporate these biological and technical processes and compare performance against alternative approaches for identifying antigen-responsive TCRs.

Several computational methods have been developed to identify antigen-responsive TCRs or infer antigen specificity from repertoire sequencing data, including approaches based on sequence similarity, motif discovery, and machine learning (12, 13). These methods address questions that are complementary to those addressed by replicateFest. In contrast to methods designed to infer antigen specificity, replicateFest focuses on statistical analysis of peptide-stimulation experiments and explicitly supports replicate-aware identification of expanded clonotypes. Because replicateFest operates on clonotype abundance measurements, it can be applied independently or integrated downstream of other antigen-specificity inference frameworks. Future studies will evaluate concordance between replicateFest and alternative methods when larger collections of experimentally validated datasets are available.

Another important future direction is incorporation of information describing similarity among peptides and TCRs. Recent methods such as tcrdist3 quantify distances between TCR sequences and identify related clonotype groups, whereas BATMAN models TCR cross-reactivity in peptide space and predicts peptide–TCR interactions using large-scale mutational datasets (7, 14). Integration of these approaches with replicateFest could improve detection of cross-reactive responses by allowing evidence to be pooled across related peptides or TCRs. As high-throughput cross-reactivity studies become more common, incorporation of peptide-space and TCR-space relationships may further improve the identification and interpretation of antigen-responsive clonotypes.

The increasing adoption of single-cell technologies that jointly measure TCR sequences and transcriptomic profiles presents an opportunity to extend the FEST analytical framework beyond clonotype abundance alone. Recent work has demonstrated that antigen-specific T-cell clonotypes can be linked to distinct transcriptional programs associated with effective antitumor immunity and response to checkpoint blockade (15). Future versions of replicateFest may integrate single-cell RNA-seq and TCR-seq data to prioritize expanded clonotypes based on transcriptional signatures of activation, memory differentiation, exhaustion, or cytotoxic function. Such multimodal integration could improve discrimination between antigen-driven expansion and non-specific proliferation while providing additional biological context for interpreting T-cell responses.

Future development will focus on expanding compatibility with single-cell TCR-seq data, integrating paired α/β chain analysis, and implementing automated thresholds for replicate concordance. By addressing these needs, replicateFest will continue to support rigorous and reproducible analysis in the rapidly evolving field of cancer immunology and beyond.

## Data Availability

The HIV-1 dataset was downloaded from the Adaptive Biotechnologies ImmuneACCESS Database, https://clients.adaptivebiotech.com/pub/chan-2020-fi. The *in silico* data is available on GitHub as a part of the replicateFest package: https://github.com/OncologyQS/replicateFest/tree/main/tests/testthat/testdata. The simulation data is available on GitHub in the paper related repository: https://github.com/OncologyQS/replicateFest_paper.
